# Introducing rotavirus vaccine in eight sub-Saharan African countries: a cost–benefit analysis

**DOI:** 10.1016/S2214-109X(21)00220-5

**Published:** 2021-07-21

**Authors:** Charles E Okafor, Obinna I Ekwunife

**Affiliations:** a Centre for Applied Health Economics, School of Medicine and Dentistry, Griffith University Nathan Campus, Nathan, QLD, Australia; b Menzies Health Institute, Griffith University Nathan Campus, Nathan, QLD, Australia; c Department of Clinical Pharmacy and Pharmacy Management, Faculty of Pharmaceutical Sciences, Nnamdi Azikiwe University, Awka, Nigeria

## Abstract

**Background:**

Stimulated by the economic challenges faced by many sub-Saharan African countries and the changes in the rotavirus burden across these countries, this study aimed to inform the decision of health policy makers of eight sub-Saharan countries, who are yet to introduce the rotavirus vaccine as of Dec 31, 2020, on the health economic consequences of the introduction of the vaccine in terms of the costs and benefits.

**Methods:**

We did a cost–benefit analysis using a simulation-based decision-analytic model for children aged younger than 1 year, who were followed up to 259 weeks, in the Central African Republic, Chad, Comoros, Equatorial Guinea, Gabon, Guinea, Somalia, and South Sudan. Data were collected and analysed between Jan 13, 2020, and Dec 11, 2020. Cost-effectiveness analysis and budget impact analysis were done as secondary analyses. Four rotavirus vaccinations (Rotarix, Rotateq, Rotavac, and Rotasiil) were compared with no vaccination. The primary outcome was disability-adjusted life-years averted, converted to monetary terms. The secondary outcomes include rotavirus gastroenteritis averted, and rotavirus vaccine-associated intussusception. The primary economic evaluation measure was the benefit–cost ratio (BCR).

**Findings:**

For the modelling period, Jan 1, 2021, to Dec 31, 2030, we found that the benefits of introducing the rotavirus vaccine outweighed the costs in all eight countries, with Chad and the Central African Republic having the highest BCR of 19·42 and 11·36, respectively. Guinea had the lowest BCR of 3·26 amongst the Gavi-eligible countries. Equatorial Guinea and Gabon had a narrow BCR of 1·86 and 2·06, respectively. Rotarix was the optimal choice for all the Gavi-eligible countries; Rotasiil and Rotavac were the optimal choices for Equatorial Guinea and Gabon, respectively.

**Interpretation:**

Introducing the rotavirus vaccine in all eight countries, but with caution in Equatorial Guinea and Gabon, would be worthwhile. With the narrow BCR for Equatorial Guinea and Gabon, cautious, pragmatic, and stringent measures need to be employed to ensure optimal health benefits and cost minimisation of the vaccine introduction. The final decision to introduce the rotavirus vaccine should be preceded by comparing its BCR to the BCRs of other health-care projects.

**Funding:**

Copenhagen Consensus Center and the Bill & Melinda Gates Foundation.

## Introduction

Gavi, the Vaccine Alliance, has supported the introduction of rotavirus vaccine in many countries.^1^ In sub-Saharan Africa, most Gavi countries (Gavi-eligible, phase 2, and phase 3 countries) and non-Gavi countries have introduced the rotavirus vaccine, and vaccine introduction is in the pipeline for some others.^1^ The descriptions of Gavi countries and non-Gavi countries are available in the appendix (p 3).

As of Dec 31, 2020, nine countries in sub-Saharan Africa are yet to introduce the rotavirus vaccine, which include the Central African Republic, Chad, Comoros, Equatorial Guinea, Gabon, Guinea, Nigeria, Somalia, and South Sudan.^1^, ^2^ Chad, the Central African Republic, Comoros, Guinea, Somalia, and South Sudan are all Gavi-eligible countries; Nigeria is in phase 2. Equatorial Guinea and Gabon are non-Gavi countries. Nigeria and the Central African Republic have received Gavi's approval with clarification on terms and conditions, and are planning the introduction of the rotavirus vaccine.^1^ South Sudan plans to apply for Gavi support to introduce the vaccine.^1^ Gabon is also planning to introduce the vaccine.^1^ No decision has been made by Chad, Somalia, Guinea, Equatorial Guinea, and Comoros on their introduction.^1^ A report from the United Nations Conference on Trade and Development predicted a 5% loss in public revenue in Africa due to COVID-19, which can affect the current and future decisions of some sub-Saharan African countries yet to introduce the rotavirus vaccine.^3^

Since 2011, the rotavirus gastroenteritis burden has been on the decrease globally.^4^ The Institute for Health Metrics and Evaluation (IHME) 2019 updates showed that most countries that have introduced the rotavirus vaccine have observed a reduction in the incidence of rotavirus gastroenteritis^4^ when compared with the 2017 reports. Notably, the Central African Republic, South Sudan, Gabon, Chad, Guinea, Equatorial Guinea, and Comoros, who are yet to introduce the rotavirus vaccine have seen a 10–30% reduction in diarrhoeal incidence rate, while Nigeria and Somalia have noted an approximate increase of 10%.^4^

**Research in context**

**Evidence before this study**

A comparison of the 2017 and 2019 Institute for Health Metrics and Evaluation reports show that while some countries that have introduced the rotavirus vaccine have observed a reduction in rotavirus gastroenteritis and mortality, some others that are yet to introduce the vaccine have also seen a reduction. We searched PubMed and Web of Science with no language restrictions for published studies from Jan 1, 2008, to June 30, 2020, on the economic evaluation of rotavirus vaccine, using the search terms (“cost effectiveness” OR “cost–benefit”) AND (“rotavirus” OR “rotavirus vaccine”). We included studies that presented both costs and outcome data; those that presented only costs data or outcome data were excluded. The findings from the studies showed that the vaccine was cost-effective in most Gavi, the Vaccine Alliance, countries and some non-Gavi countries. There was no available study that did a cost–benefit analysis using the primary health outcome, disability-adjusted life-years (DALYs) averted, but with a secondary outcome, rotavirus gastroenteritis health-care cost averted. Also, most available studies did not account for the effect of rotavirus vaccine-associated intussusception on the costs and benefits. Although cost-effectiveness analysis is a useful tool for decision making by health policy makers, it might not be sufficient because an intervention could be cost-effective but not cost-beneficial. In sub-Saharan Africa, which has most of the poorest countries in the world, making health-care decisions with cost–benefit analysis is becoming more relevant than cost-effectiveness analysis. Cost–benefit analysis has a greater advantage in the allocation of budgets across a range of projects by ranking the projects' benefit–cost ratios. With the current financial implications of the COVID-19 pandemic in sub-Saharan Africa, the importance of cost–benefit analysis to guide health decisions cannot be overemphasised. Furthermore, changes in the disease burden in countries yet to introduce the vaccine might affect the cost–benefit or cost-effectiveness of the vaccine introduction.

**Added value of this study**

This study was a cost–benefit analysis and, to the best of our knowledge, is the first to provide the benefit–cost ratio of introducing the rotavirus vaccine. We estimated the total benefit as the sum of the monetary value of the DALY averted, and the rotavirus gastroenteritis health-care cost averted. This study is also the first to evaluate the economic implications of rotavirus vaccine introduction in Equatorial Guinea and Gabon. Furthermore, our study shows the optimal vaccines to use for each of the eight countries: Central African Republic, Chad, Comoros, Equatorial Guinea, Gabon, Guinea, Somalia, and South Sudan.

**Implication of all the available evidence**

The results of our study provide further evidence that introducing the rotavirus vaccine, especially in Gavi-eligible countries yet to introduce the vaccine, will be a worthwhile decision. The study also underscores the need for most non-Gavi countries to employ cautious, pragmatic, and stringent measures when introducing and sustaining the vaccine in their health-care immunisation system. The final decision to introduce the rotavirus vaccine should be preceded by comparing its benefit–cost ratio to that of other health-care projects.

Among the economic challenges faced by many sub-Saharan African countries, which have worsened the already existing scarcity of financial resources, and the changes in the disease burden across countries, this study aimed to inform the decisions of the health policy makers of countries in sub-Saharan African that are yet to introduce the rotavirus vaccine on the health economic consequences of the introduction in terms of the costs and benefits. The outcome of this study will help to prioritise the rotavirus vaccine introduction project relative to other health-care projects previously evaluated by comparing their benefit–cost ratios (BCRs).

## Methods

### Study design

This study was a cost–benefit analysis that followed the Harvard-led guideline^5^ for cost–benefit analysis, and the International Society for Pharmacoeconomics and Outcome Research Consolidated Health Economic Evaluation Reporting Standards guideline.^6^ The Harvard-led guideline was built on the existing International Decision Support Initiative reference case, which discusses the general framework for economic evaluation and the conduct of cost-effectiveness analyses to meet the informational needs of decision makers, especially in low-income and middle-income countries.^5^

**Figure 1:**
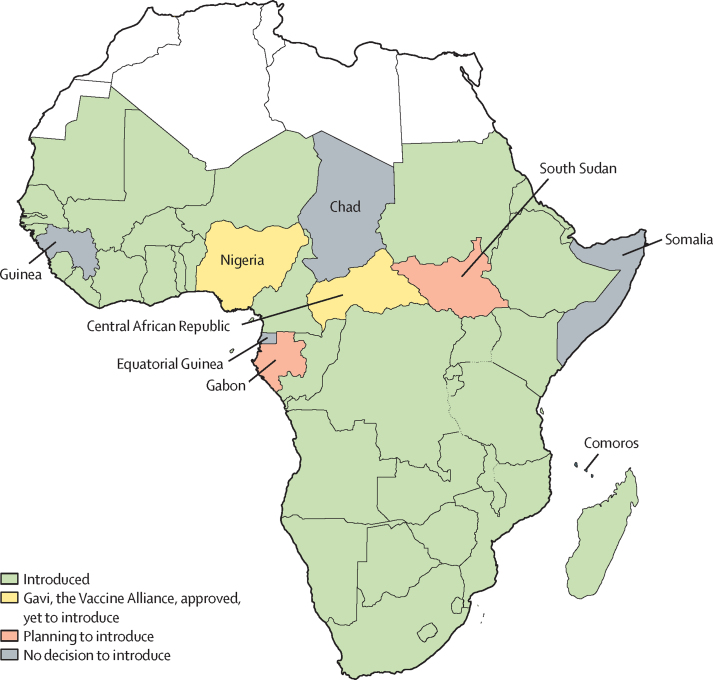
Map of sub-Saharan Africa showing countries introduction status of rotavirus vaccine as of 2020

The study was done for children aged younger than 1 year, who were followed up for up to 259 weeks in the model (ie, <5 years) to accumulate the associated cost and benefit of the vaccine, rotavirus gastroenteritis, and rotavirus vaccine-associated intussusception. Eight sub-Saharan African countries were used in the study: the Central African Republic, Chad, Comoros, Equatorial Guinea, Gabon, Guinea, Somalia, and South Sudan;^7^ Nigeria was excluded in this assessment because a study on the introduction of the rotavirus vaccine in Nigeria already exists.^8^ Data were collected and analysed between Jan 13, 2020, and Dec 11, 2020. Figure 1 presents a map of sub-Saharan Africa, showing the eight countries introduction status of rotavirus vaccine as of 2020.

The study was done from the perspective of the payer (government). For Gavi-eligible countries, the payer's perspective includes the co-financing share of the government for the vaccine and the immunisation delivery costs spent by the government to deliver the vaccine to the children. For non-Gavi countries, the costs are fully funded by the government without Gavi support.

### Comparators

Four vaccine scenarios were compared with a control (ie, no vaccine). First, vaccination with Rotarix, a live monovalent human attenuated oral rotavirus vaccine (RV1) manufactured by GlaxoSmithKline Biologicals (Belgium). Rotarix presentation is a liquid single-dose pack in a plastic tube. Two doses of Rotarix are required for full immunisation. Second, vaccination with Rotateq (for non-Gavi countries only), a live pentavalent, human–bovine reassortant vaccine (RV5) manufactured by Merck & Co (Kenilworth, NJ, USA). Rotateq presentation is a liquid single-dose pack in a plastic tube. Three doses of Rotateq are required for full immunisation. Rotateq was not considered as a comparator for the Gavi-eligible countries because the manufacturer has withdrawn supplies to Gavi countries.^9^ Third, vaccination with Rotavac, a live monovalent human-bovine liquid frozen oral rotavirus vaccine (RV1), manufactured by Bharat Biotech (Hyderabad, India). Rotavac presentation is a liquid (frozen), five-dose pack in a vial. Three doses of Rotavac are required for full immunisation. Fourth, vaccination with Rotasiil, a live pentavalent bovine-human reassortant oral rotavirus vaccine (RV5), manufactured by the Serum Institute (Pune, India). Rotasiil presentation is in lyophilised form, a two-dose pack in a vial. Three doses of Rotasiil are required for full immunisation.

### Model and assumptions

**Figure 2:**
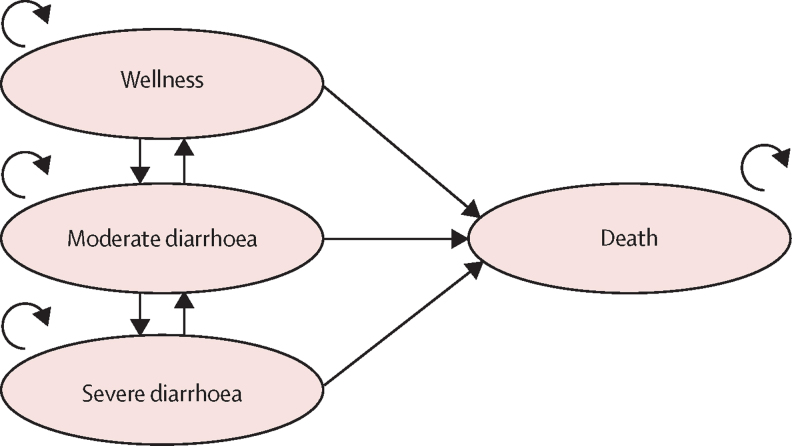
Model figure of the health states and the possible transitions The ovals indicate the health states; the straight arrows indicate the possibility of transition to the different health states; and the curly arrows indicate the possibility of remaining in the different health states.

This study was a Markov model-based study. The states in the model include wellness, moderate diarrhoea, severe diarrhoea, and death. As per the vaccination age eligibility for rotavirus vaccine, the starting population used in each model was children aged younger than 1 year. Also, based on the vaccination coverage of each country, the vaccinated children were followed up to 259 weeks (ie, <5 years) years to estimate the associated costs and benefits of the rotavirus vaccination. Each model was built to have 260-week cycles. Ten cohorts from 2021 to 2030 were studied. Figure 2 describes the model. The transition probabilities of moving to, and remaining in each health states, life table data, and the disability weights for moderate and severe diarrhoea were obtained from the 2019 IHME report for each country and a systematic review.^4^, ^10^

The model estimated the number of moderate and severe rotavirus gastroenteritis cases and the number of rotavirus gastroenteritis deaths for the cohort in each country. We assumed that moderate rotavirus gastroenteritis will be managed as outpatient cases, whereas severe rotavirus gastroenteritis cases will be managed by hospital admissions. We also estimated the number of rotavirus vaccine-associated intussusception and deaths from the incidence rate, case-fatality rate, and relative risk of RV1 and RV5 vaccines.^4^, ^11^, ^12^ Details of the input parameters are shown in table 1, table 2, and the appendix (pp 1–2).

**Table 1:** Heterogenous input variables in the model

		**Central African Republic**	**Chad**	**Comoros**	**Equatorial Guinea**	**Gabon**	**Guinea**	**Somalia**	**South Sudan**
Crude birth rate per 1000 population^7^		35·35	42·17	31·88	33·24	31·61	36·36	41·75	35·01
Total population in 2019, millions^7^		4·75	15·95	0·85	1·36	2·17	12·77	15·44	11·06
Under-1-year population in 2019^7^		167 116	669 774	27 232	44 600	68 800	462 470	637 304	389 456
Weekly transition probabilities, ×10^−3^									
	Wellness to moderate diarrhoea^4^	45·96 (37·66–53·86)	53·48 (47·61–58·26)	41·59 (32·91–50·14)	31·30 (23·88–39·49)	33·92 (25·48–40·82)	30·22 (24·07–37·36)	42·44 (33·90–51·08)	46·11 (37·38–54·22)
	Moderate to wellness diarrhoea^4^	987·26 (984·13–990·07)	986·59 (984·27–988·79)	989·69 (987·18–992·05)	991·28 (986·34–994·70)	991·46 (989·18–993·66)	991·28 (988·71–993·32)	989·70 (987·15–991·98)	987·90 (985·29–990·35)
	Moderate to severe diarrhoea^4^	4·85 (3·53–6·50)	4·35 (3·23–5·71)	3·56 (2·64–4·63)	3·64 (1·45–7·18)	3·12 (2·28–4·25)	3·57 (2·59–4·91)	3·17 (2·37–4·20)	4·40 (3·44–5·58)
	Severe to moderate diarrhoea^4^	998·79 (998·4–999·1)	998·89 (998·54–999·14)	999·37 (999·17–999·53)	999·41 (998·91–999·71)	999·53 (999·36–999·66)	999·16 (998·91–999·37)	999·18 (998·92–999·39)	999·05 (998·80–999·26)
	Recurrent moderate diarrhoea^4^, ^10^	7·22 (5·89–8·49)	8·43 (7·48–9·20)	6·52 (5·14–7·89)	4·89 (3·72–6·19)	5·30 (3·97–6·40)	4·71 (3·75–5·84)	6·66 (5·30–8·04)	7·24 (5·85–8·55)
	Recurrent severe diarrhoea^4^, ^10^	0·54 (0·39–0·72)	0·48 (0·36–0·64)	0·40 (0·29–0·52)	0·40 (0·16–0·80)	0·35 (0·25–0·47)	0·40 (0·29–0·55)	0·35 (0·26–0·47)	0·49 (0·38–0·62)
	Remaining well^4^, ^10^	953·37 (945·26–961·83)	945·89 (940·92–966·92)	958·18 (949·56–966·92)	968·51 (960·22–975·99)	965·96 (959·00–974·43)	969·34 (962·10–975·59)	957·09 (948·31–965·75)	953·43 (945·20–962·26)
	All-cause under-5 mortality^4^	0·55 (0·44–0·69)	0·49 (0·41–0·60)	0·21 (0·16–0·26)	0·15 (0·11–0·21)	0·1 (0·09–0·17)	0·41 (0·33–0·50)	0·42 (0·32–0·53)	0·42 (0·34–0·51)
	All-cause under-5 diarrhoea to death^4^	0·12 (0·07–0·19)	0·14 (0·09–0·22)	0·02 (0·01–0·04)	0·04 (0·02–0·08)	0·001 (0·0003–0·002)	0·03 (0·01–0·04)	0·05 (0·03–0·08)	0·04 (0·02–0·07)
Intussusception									
	Incidence per 10 000 population aged under 1 year without rotavirus vaccine^11^	34 (13–56)	34 (13–56)	34 (13–56)	34 (13–56)	34 (13–56)	34 (13–56)	34 (13–56)	34 (13–56)
	Case-fatality without rotavirus vaccine^4^, ^11^, ^12^	10·1% (7·24–17·78)	10·1% (7·24–17·78)	2·02% (1·45–3·56)	0·42% (0·30–0·73)	0·21% (0·15–0·37)	2·02% (1·45–3·56)	5·05% (3·62–8·89)	3·03% (2·17–5·33)
	Risk ratio of first dose, RV1^12^	4·70 (2·6–8·4)	4·70 (2·6–8·4)	4·70 (2·6–8·4)	4·70 (2·6–8·4)	4·70 (2·6–8·4)	4·70 (2·6–8·4)	4·70 (2·6–8·4)	4·70 (2·6–8·4)
	Risk ratio of second dose, RV1^12^	1·80 (1·30–2·60)	1·80 (1·30–2·60)	1·80 (1·30–2·60)	1·80 (1·30–2·60)	1·80 (1·30–2·60)	1·80 (1·30–2·60)	1·80 (1·30–2·60)	1·80 (1·30–2·60)
	Risk ratio of third dose, RV1 or RV5^12^	1·14 (0·75–1·74)	1·14 (0·75–1·74)	1·14 (0·75–1·74)	1·14 (0·75–1·74)	1·14 (0·75–1·74)	1·14 (0·75–1·74)	1·14 (0·75–1·74)	1·14 (0·75–1·74)
	Risk ratio of first dose, RV5^12^	6·10 (3·00–12·10)	6·10 (3·00–12·10)	6·10 (3·00–12·10)	6·10 (3·00–12·10)	6·10 (3·00–12·10)	6·10 (3·00–12·10)	6·10 (3·00–12·10)	6·10 (3·00–12·10)
	Risk ratio of second dose, RV5^12^	1·70 (1·10–2·60)	1·70 (1·10–2·60)	1·70 (1·10–2·60)	1·70 (1·10–2·60)	1·70 (1·10–2·60)	1·70 (1·10–2·60)	1·70 (1·10–2·60)	1·70 (1·10–2·60)
	Cost of intussusception management per case, US$^13^	$150·73	$118·10	$143·09	$145·85	$138·91	$102·46	$89·39	$138·39
	Vaccination coverage^14^	47%	41%	91%	25%	70%	45%	42%	49%
	Estimated vaccination service points^15^, ^16^	700	1016	50	70	250	1500	1300	800
	Diarrhoeal symptoms caused by rotavirus^4^	45%	40%	40%	50%	30%	40%	50%	40%
	Diarrhoea death caused by rotavirus^4^	30%	25%	27%	32%	15%	27%	33%	26%
Vaccine wastage									
	Rotarix^15^	4%	4%	4%	4%	4%	4%	4%	4%
	Rotateq^15^	..	..	..	3%	3%	..	..	..
	Rotavac^15^	49%	26%	20%	50%	38%	41%	34%	25%
	Rotasiil^15^	20%	10%	9%	20%	15%	16%	14%	10%
Waste-adjusted cold chain volume per fully immunised child, cm^3^									
	Rotarix^9^	36·00	36·00	36·00	36·00	36·00	36·00	36·00	36·00
	Rotateq^9^	..	..	..	137·70	137·70	..	..	..
	Rotavac^9^	37·50	18·90	18·00	38·60	27·20	35·60	27·80	18·00
	Rotasiil^9^	70·20	35·10	35·00	70·30	53·50	54·00	53·60	35·10
Personnel cost per fully immunised child, US$									
	Rotarix delivery^15^, ^17^	$1·13 (0·85–1·41)	$1·17 (0·88–1·46)	$1·95 (1·46–2·44)	$3·03 (2·36–3·94)	$2·58 (1·94–3·23)	$1·41 (1·06–1·76)	$0·93 (0·70–1·16)	$1·83 (1·37–2·29)
	Rotateq delivery^15^, ^17^	..	..	..	$5·54 (4·16–6·92)	$4·83 (3·62–6·04)	..	..	..
	Rotavac delivery^15^, ^17^	$1·71 (1·31–2·19)	$1·76 (1·32–2·20)	$2·72 (2·04–3·40)	$4·05 (3·02–5·06)	$3·30 (2·51–4·19)	$1·93 (1·45–2·41)	$1·33 (1·00–1·66)	$2·51 (1·88–3·14)
	Rotasiil delivery^15^, ^17^	$1·71 (1·28–2·14)	$1·77 (1·34–2·28)	$2·95 (2·26–3·78)	$4·20 (3·30–5·51)	$3·47 (2·58–4·29)	$2·11 (1·58–2·64)	$1·40 (1·07–1·79)	$2·60 (1·93–3·23)
Logistic cost per fully immunised child, US$									
	Rotarix delivery^15^, ^17^	$0·31 (0·23–0·39)	$0·29 (0·20–0·37)	$0·43 (0·32–0·54)	$0·55 (0·41–0·69)	$0·46 (0·34–0·58)	$0·33 (0·25–0·41)	$0·25 (0·18–0·31)	$0·37 (0·28–0·46)
	Rotateq delivery^15^, ^17^	..	..	..	$0·61 (0·46–0·76)	$0·48 (0·36–0·60)	..	..	..
	Rotavac delivery^15^, ^17^	$0·33 (0·28–0·42)	$0·22 (0·16–0·28)	$0·37 (0·28–0·46)	$0·45 (0·34–0·56)	$0·39 (0·29–0·50)	$0·29 (0·21–0·36)	$0·23 (0·17–0·29)	$0·28 (0·22–0·35)
	Rotasiil delivery^15^, ^17^	$0·38 (0·29–0·48)	$0·36 (0·27–0·45)	$0·41 (0·30–0·51)	$0·60 (0·45–0·75)	$0·46 (0·35–0·58)	$0·32 (0·24–0·40)	$0·28 (0·21–0·35)	$0·37 (0·27–0·46)
Waste-adjusted immunisation delivery cost per fully immunised child, US$									
	Rotarix delivery^15^, ^17^	$1·44 (1·08–1·80)	$1·46 (1·10–1·83)	$2·38 (1·79–2·98)	$3·58 (2·69–4·48)	$3·04 (2·28–3·80)	$1·74 (1·31–2·18)	$1·18 (0·89–1·48)	$2·20 (1·65–2·75)
	Rotateq delivery^15^, ^17^	..	..	..	$6·15 (4·61–7·69)	$5·31 (3·98–6·64)	..	..	..
	Rotavac delivery^15^, ^17^	$2·04 (1·53–2·55)	$1·98 (1·49–2·48)	$3·09 (2·32–3·86)	$4·50 (3·38–5·63)	$3·69 (2·77–4·61)	$2·22 (1·67–2·78)	$1·56 (1·17–1·95)	$2·79 (2·09–3·49)
	Rotasiil delivery^15^, ^17^	$2·10 (1·58–2·63)	$2·13 (1·60–2·66)	$3·36 (2·52–4·20)	$4·80 (3·60–6·00)	$3·93 (2·95–4·91)	$2·43 (1·82–3·04)	$1·68 (1·26–2·10)	$2·97 (2·23–3·71)
	International handling (as a percentage of vaccine cost)^18^	3·50%	3·50%	3·50%	4·00%	4·00%	3·50%	3·50%	3·50%
	Freight (as a percentage of vaccine cost)^18^	7·50%	7·50%	7·50%	8·00%	8·00%	7·50%	7·50%	7·50%
Life expectancy, years									
	Under 1^4^	52·35	60·38	68·73	65·98	67·81	61·24	58·52	63·74
	1–4^4^	55·90	63·75	70·62	66·89	68·39	63·86	61·51	67·20
GDP per capita, US$^19^		$468	$710	$1394	$8132	$7667	$1064	$420*	$1120†
GNI per capita, US$^19^		$520	$700	$1420	$6460	$7210	$950	$395*	$1090†
GNI per capita PPP, International $^19^		$1060	1620	$3210	$14 640	$14 350	$2650	$1116*	$1080†
DALY to GNI multiplier^5^		0·77	$0·83	1·03	2·28	2·20	1·05	0·81	0·64
Cost of moderate RVGE management, US$^20^		$4·98	$4·98	$5·41	$8·20	$8·20	$4·98	$4·98	$4·98
Cost of severe RVGE management, US$^20^		$53·25	$53·25	$57·68	$86·46	$86·46	$53·25	$53·25	$53·25
Care-seeking at health facilities^21^		32·60%	32·60%	41·50%	32·60%	32·60%	42·40%	41·50%	41·50%

Data are n, US$, %, or (95% CI). GDP=gross domestic product. GNI=gross national income. DALY=disability-adjusted life-years. RV1=monovalent rotavirus vaccine. RV5=pentavalent rotavirus vaccine. RVGE=rotavirus gastroenteritis. PPP=power purchasing parity.

* Adjusted from 2011 values.

† 2015 value.

**Table 2:** Homogenous input variables in the model

		**Value**	**Distribution**
Price per dose, US$			
	Rotarix (Gavi countries)^2^	2·29	*..*
	Rotarix (non-Gavi countries)^2^	8·05	*
	Rotateq (non-Gavi countries)^2^	11·15	*
	Rotavac (Gavi countries) ^2^	0·85	*..*
	Rotavac (non-Gavi countries) ^2^	3·10	*
	Rotasiil (Gavi countries) ^2^	0·95	*..*
	Rotasiil (non-Gavi countries) ^2^	3·35	*
	Co-financing share for initial self-financing countries for Rotarix, US$^22^	$0·20	*..*
	Co-financing share for initial self-financing countries for other vaccines, US$^22^	$0·13	*..*
Vaccine effectiveness†			
	First dose Rotarix^23^	0·26	0·25 to 0·58
	Second dose Rotarix^23^	0·59	0·37 to 0·73
	First dose Rotateq^23^	0·44	0·17 to 0·61
	Second dose Rotateq^23^	0·47	0·18 to 0·65
	Third dose Rotateq^23^	0·49	0·19 to 0·68
	First dose Rotavac^24^	0·23	0·21 to 0·53
	Second dose Rotavac^24^	0·34	0·30 to 0·62
	Third dose Rotavac^24^	0·56	0·37 to 0·70
	First dose Rotasiil^25^, ^26^	0·20	0·19 to 0·48
	Second dose Rotasiil^25^, ^26^	0·30	0·22 to 0·56
	Third dose Rotasiil^25^, ^26^	0·50	0·28 to 0·66
	Effectiveness after 2 weeks^27^	0·66	0·48 to 0·81
	Effectiveness after 4 weeks^27^	0·62	0·47 to 0·75
	Effectiveness after 8 weeks^27^	0·57	0·45 to 0·67
	Effectiveness after 12 weeks^27^	0·54	0·44 to 0·64
	Effectiveness after 24 weeks^27^	0·49	0·40 to 0·61
	Effectiveness after 36 weeks^27^	0·46	0·33 to 0·60
	Effectiveness after 48 weeks^27^	0·44	0·27 to 0·59
	Effectiveness after 72 weeks^27^	0·41	0·17 to 0·58
	Effectiveness after 96 weeks^27^	0·38	0·09 to 0·58
	Effectiveness after 144 weeks^27^	0·35	−0·04 to 0·57
	Effectiveness after 192 weeks^27^	0·32	−0·14 to 0·57
Disability weights‡			
	Moderate diarrhoea^4^	0·051	0·032 to 0·074
	Severe diarrhoea^4^	0·133	0·088 to 0·190
	Intestinal obstruction^4^	0·324	0·220 to 0·442
Discount rate			
	Cost^28^	5%	NA (min 0%, max 10%)
	Utility^28^	5%	NA (min 0%, max 10%)
Full immunisation doses (Rotarix)^9^		2	*..*
Full immunisation doses (Rotateq, Rotavac, and Rotasiil)^9^		3	*..*
Reserve stock^29^		25%	..

Data are mean and interval for price per dose; mean and 95% CI for vaccine effectiveness and disability weights; % for discount rate and reserve stock; and n for full immunisation doses. NA=not applicable.

* The distribution of these data are ±25% interval.

† Log-normal distribution.

‡ Beta distribution.

### Time horizon, inflation, discount rate, and currency

The costs and outcomes were simulated within 260 weeks. A discount rate of 5% for low-income and middle-income countries was used in the costs and outcomes analysis.^28^ The costs were inflated, where applicable, as per guidelines of the Campbell and Cochrane Economics Methods Group and the Evidence for Policy and Practice Information and Coordinating Centre.^30^ All costs were presented in 2019 US$ values.

### Vaccine coverage, effectiveness, and wastage rate

We applied the diphtheria-tetanus-pertussis coverage rate for each country in the estimation of the costs and outcomes.^14^ The effectiveness of the first and second dose for Rotarix, and the effectiveness of the first, second, and third dose for Rotateq were obtained from a systematic review for countries with high diarrhoeal mortality.^23^ For Rotavac and Rotasiil, their clinical efficacy data were used for the first, second, and third doses.^24^, ^25^, ^26^ The first dose of all the vaccines were administered to children aged 6 weeks; the second dose of Rotateq, Rotavac, and Rotasiil were administered to children aged 16 weeks; and the second dose of Rotarix and the third dose of the other vaccines were administered to children aged 24 weeks in the model. The vaccines waning effect after vaccination were captured in the model.^27^

The WHO vaccine wastage rates calculator was used to estimate the wastage rate for Rotarix (one dose per tube pack size), Rotateq (one dose per tube pack size), Rotavac (five doses per vial pack size), and Rotasiil (two doses per vial pack size) from the year 2021 to 2030 for each country.^15^ The major covariates for vaccine wastage rate include the number of doses per tube or vial, vaccination coverage rate, and the number of service points. We estimated the number of service points per 10 000 population per country from their number of health workforce availability per 10 000 population, which was applied to estimate the wastage rates.^15^, ^16^ Details are presented in table 1.

### Estimation of the costs

We adopted a guideline for estimating the costs of introducing new vaccines into the national immunisation system.^29^ The total vaccine cost comprises the unadjusted cost of the vaccine for full immunisation, vaccine wastage cost, and international handling and freight cost for each vaccination scenario. The immunisation delivery cost comprises the personnel cost and logistic cost. The personnel cost includes the salaries to health-care professionals, health assistants, administrative and other supporting staff, advocacy and social mobilisation cost, and surveillance cost. The logistic cost includes the waste-adjusted cost of storing vaccine in the cold chain, vaccine transportation, buildings (structures and offices), vehicles and its maintenance, safety boxes, syringes, and training of immunisation staff.

We used UNICEF supply price per dose for Rotarix (US$2·29), Rotavac ($0·85), and Rotasiil ($0·95) as vaccines' prices for the Gavi-eligible countries. The median supply price by vaccine manufacturers to upper-middle-income countries was used for Equatorial Guinea and Gabon, the two non-Gavi countries in this study.^2^ A full immunisation co-financing share of $0·4 from the vaccine prices was used as the unadjusted vaccine cost for the Central African Republic, Chad, Guinea, Somalia, and South Sudan based on Gavi funding guidelines for countries at the initial self-financing phase, which translates to $0·20 per dose for Rotarix and $0·13 per dose for Rotavac and Rotasiil.^22^ The starting fraction for Comoros (phase 1 country) is 18·2%, which was used as their price fraction in the year 2021. From 2022 to 2030, Comoros price fraction will increase annually by 15% of the previous year's fraction. The vaccine co-financing share for Comoros is their price fraction (for each year) multiplied by the co-financed vaccine price.^2^, ^22^ The UNICEF international handling cost and freight cost for new vaccines for least and non-least developed countries were applied.^18^ A 25% reserve stock was applied in the first year in calculating the annual vaccine cost.

The immunisation delivery costs were estimated from the immunisation delivery cost catalogue of the Immunisation Costing Action Network (ICAN) based on the vaccination coverage, population, price-level ratio, and gross domestic product (GDP) per capita of each country.^17^, ^19^ A detailed description of how the immunisation delivery costs were estimated from the ICAN review based on these covariates is available in the appendix (p 3).

The costs of moderate and severe rotavirus gastroenteritis management at health-care facilities for low-income, lower-middle-income, and upper-middle-income countries were obtained from a study that estimated the costs of rotavirus gastroenteritis in low-income and middle-income countries,^20^ and based on diarrhoeal health-care seeking in western, central, and eastern Africa.^21^ Due to the low health-care seeking in sub-Saharan Africa, for moderate diarrhoea cases, we assumed that for the low-income countries, only a quarter of their respective health-care seeking proportions will visit the outpatient health-care facilities, whereas for the middle-income countries, we assumed that only half of their respective health care-seeking proportions will visit the outpatient health-care facilities (table 1).

The cost of managing rotavirus vaccine-associated intussusception was estimated from a Nigerian-based study.^30^ The study showed that the non-operative management that applies hydrostatic reduction under ultrasound guidance is very effective and has low risk of mortality.^13^ Given the high cost of management by surgical approach, the associated risk, and poverty in sub-Saharan Africa, we assumed all patients will be managed by the non-operative procedure. The cost estimate from the study was adjusted to the cost for the respective countries based on their relative price-level ratios.^19^ Details of the cost components are shown in table 1.

### Outcomes

Disability-adjusted life-years (DALYs) averted was the primary health outcome measure, which was calculated as the sum of the averted years lived with disability (YLD) from morbidity and the averted years of life lost (YLL) from mortality.^31^ Our primary decision analysis was cost–benefit analysis. Cost-effectiveness analysis and budget impact analysis were done as secondary analyses to provide additional evidence to the primary results.

The YLD was estimated as:

YLD=number of cases×duration till remission or death×disability weight

The YLL was estimated as:

YLD=number of deaths due to rotavirus gastroenteritis×life expectancy at the age of death

The DALY for each vaccine scenario was inclusive of the DALY due to rotavirus vaccine-associated intussusception. We used disability weight for intestinal obstruction as disability weight for intussusception.^4^ The DALY averted was used in the be BCR estimation. The BCR valuation was based on the value of statistical life-year.^5^ Using the Harvard-led approach, the value of statistical life (VSL) to gross national income (GNI) per capita power purchasing parity (PPP) multiplier for the reference case was approximately 160 times.^5^ We estimated the VSL to GNI per capita PPP multiplier for each country using an income elasticity of 1·5, the 2019 GNI per capita PPP for the reference case and the GNI per capita PPP for the target country using the formula:^5^

$$(\frac{{\text{VSL}}_{\text{target}}}{{\text{Income}}_{\text{target}}})=(\frac{{\text{VSL}}_{\text{reference}}}{{\text{Income}}_{\text{reference}}})\times {\left(\frac{{\text{Income}}_{\text{target}}}{{\text{Income}}_{\text{reference}}}\right)}^{\left(\text{elasticity}-1\right)}$$

The VSL year was estimated by dividing the VSL by the average life expectancy of an average aged adult which is proxied to be half the life expectancy at birth. For example, the life expectancy at birth in Chad is 60·38 years, which translates to a 30·19-year life expectancy of an adult of average age, with a VSL to GNI multiplier of 25·05.^4^ The YLL component of the DALY averted will, therefore, be valued with a factor of 0·83 times (25·05 divided by 30·19) the GNI per capita. The 2019 IHME report reveals that the YLL component is over 95% of the total DALY for diarrhoeal diseases in sub-Saharan Africa, which implies that the YLD component will contribute to less than 5% of the total DALY.^4^ The YLD was also valued with the same YLL factor for parsimony. Thus, one DALY averted was valued at 0·83 times the GNI per capita of Chad.

The total benefit was estimated as the sum of the monetary value of the DALY averted and the rotavirus gastroenteritis health-care cost averted. The threshold forthe BCR analysis was 1. A conservative threshold of less than 1 times the GDP per capita of a country was applied in the cost-effectiveness analysis.^32^

### Handling uncertainty

The appropriate distribution for each variable was used in the models,^33^ and the simultaneous uncertainty in the variables was assessed using probabilistic sensitivity analysis. We ran 1000 iterations of the Monte-Carlo simulation for each scenario. We also did a deterministic sensitivity analysis to identify key parameters that could affect the results. The analysis was done using Microsoft Excel 365.

### Role of the funding source

The funder of the study had no role in the study design, data collection, data analysis, data interpretation, or writing of the manuscript.

## Results

For the modelling period, Jan 1, 2021, to Dec 31, 2030, we found that for all Gavi-eligible countries, vaccination with Rotarix had the lowest cost followed by Rotavac, except for the Central African Republic, where there was a marginal cost difference between Rotavac and Rotasiil. For the Gavi-eligible countries, the cost of vaccination with Rotarix per child ranged from US$1·52 for Somalia to $3·27 for Comoros. Rotasiil had the lowest cost for Equatorial Guinea ($13·12) and Gabon ($12·33), followed by Rotavac.

The DALY averted was optimal with Rotarix, followed by Rotavac for all the Gavi-eligible countries. However, Rotateq followed by Rotarix had the optimal DALY averted for Equatorial Guinea, while Rotarix followed by Rotateq was optimal for Gabon. The highest DALY was averted in Chad (170 360 DALYs), while the lowest was observed in Equatorial Guinea (175 DALYs) for the target population. The economic impact of the optimal vaccine in saving the cost of rotavirus gastroenteritis management ranged from US$172 107 in Equatorial Guinea to $2 104 677 in Somalia for the 10 years. Equatorial Guinea had the lowest rotavirus vaccine-associated intussusception (335 children) and deaths (one child), whereas Chad had the highest cases (5738 children), and deaths (594 children). The risk of intussusception after vaccination was not higher than the background risk of intussusception in all the countries.

For all the Gavi-eligible countries, Rotarix offered the highest BCR followed by Rotavac compared with the other vaccines. Rotasiil offered the highest BCR for Equatorial Guinea, whereas Rotavac offered the highest for Gabon. Chad had the highest BCR compared to all the other countries (19·42), followed by the Central African Republic (11·36), whereas Equatorial Guinea had the lowest BCR (1·86) followed by Gabon (2·06). Based on the BCR threshold of 1, the rotavirus vaccine is worth introducing in the eight countries.

**Figure 3:**
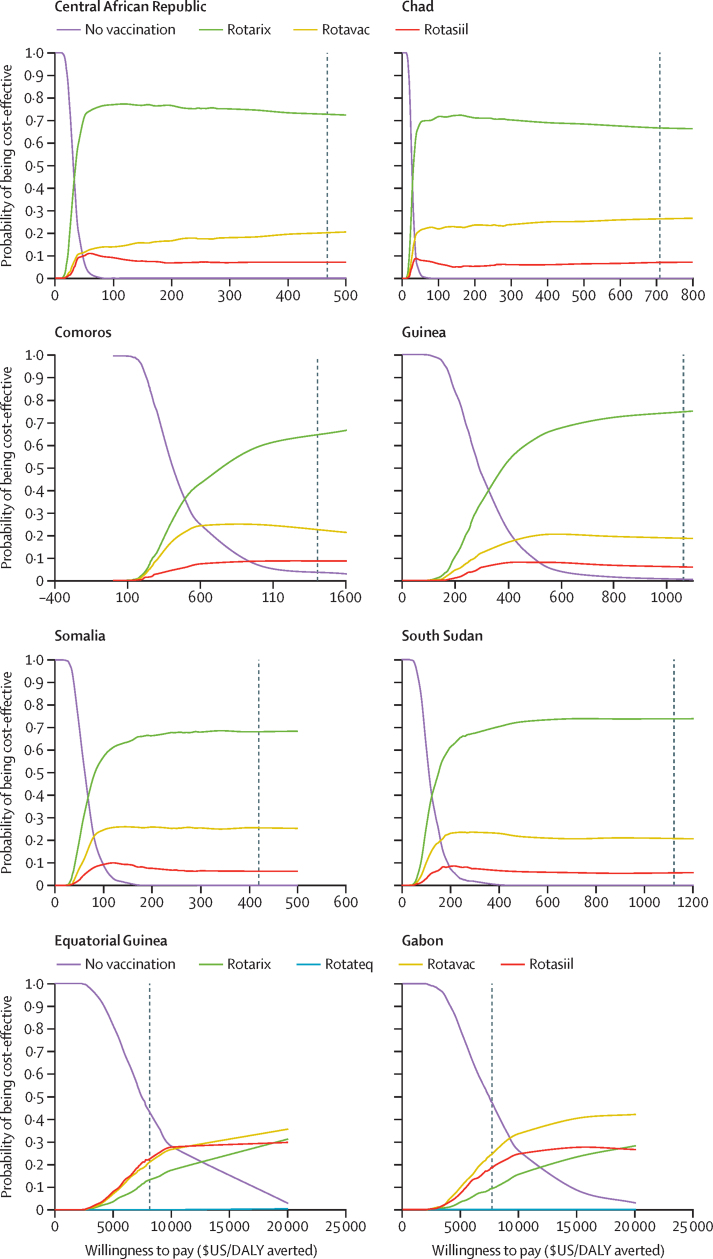
Cost-effectiveness acceptability curves of the interventions for the respective countries The broken dotted lines represent the threshold of 1 times the gross domestic product per capita for each country. DALY=disability-adjusted life-year.

Rotarix, Rotavac, and Rotasiil were cost-effective for all the Gavi-eligible countries. The average cost-effectiveness ratios (ACER) were below the threshold, which ranged from US$27·53 per DALY averted in Chad to $445·16 per DALY averted in Comoros. However, going by the threshold, the rotavirus vaccines were not cost-effective for Equatorial Guinea (ACER $8265 per DALY averted) and Gabon (ACER $7828 per DALY averted). The incremental cost-effectiveness ratio (ICER) results showed that Rotarix and Rotavac were dominant when compared with Rotasiil for all Gavi-eligible countries, with Rotarix being more dominant. Thus, Rotarix is the optimal vaccine for all Gavi-eligible countries. Figure 3 presents the cost-effectiveness acceptability curves of the interventions for each country.

The rotavirus gastroenteritis health-care cost averted, and intussusception health-care cost spent will create a net positive impact of about 17% (in Guinea) to 41% (in Somalia) on the total vaccination cost for the Gavi-eligible countries and about 9% and 5% effect for Equatorial Guinea and Gabon, respectively. Details of the results are available in table 3.

**Table 3:** Discounted costs and outcomes of the vaccination from the probabilistic sensitivity analysis from the year 2021 to 2030 for ten cohorts

	**Central African Republic**	**Chad**	**Comoros**	**Equatorial Guinea**	**Gabon**	**Guinea**	**Somalia**	**South Sudan**
**Median vaccination cost per child, US$**								
Rotarix vaccine	$0·67 (0·65–0·69)	$0·68 (0·66–0·69)	$1·49 (1·46–1·52)	$13·60 (13·32–13·78)	$13·61 (13·36–13·86)	$0·70 (0·68–0·71)	$0·66 (0·65–0·67)	$0·67 (0·66–0·68)
Rotarix delivery	$1·05 (1·03–1·08)	$1·07 (1·05–1·09)	$1·78 (1·75–1·81)	$2·62 (2·57–2·67)	$2·21 (2·17–2·25)	$1·28 (1·26–1·30)	$0·86 (0·85–0·88)	$1·60 (1·57–1·63)
Rotateq vaccine	..	..	..	$28·25 (27·77–28·73)	$28·14 (27·66–28·62)	..	..	..
Rotateq delivery	..	..	..	$4·49 (4·41–4·57)	$3·81 (3·74–3·88)	*..*	*..*	..
Rotavac vaccine	$0·65 (0·64–0·67)	$0·58 (0·57–0·59)	$0·92 (0·90–0·94)	$11·22 (10·95–11·35)	$10·21 (10·03–10·39)	$0·62 (0·61–0·63)	$0·60 (0·59–0·61)	$0·56 (0·55–0·57)
Rotavac delivery	$1·52 (1·49–1·55)	$1·45 (1·42–1·48)	$2·29 (2·25–2·33)	$3·27 (3·21–3·33)	$2·69 (2·64–2·74)	$1·62 (1·59–1·65)	$1·07 (1·05–1·09)	$1·99 (1·95–2·03)
Rotasiil vaccine	$0·59 (0·57–0·60)	$0·55 (0·54–0·56)	$0·95 (0·93–0·97)	$9·59 (9·43–9·75)	$9·28 (9·12–9·44)	$0·57 (0·56–0·58)	$0·56 (0·55–0·57)	$0·55 (0·54–0·56)
Rotasiil delivery	$1·55 (1·52–1·58)	$1·58 (1·55–1·61)	$2·47 (2·42–2·52)	$3·53 (3·47–3·59)	$2·89 (2·84–2·94)	$1·73 (1·73–1·79)	$1·23 (1·21–1·25)	$2·17 (2·13–2·21)
**Vaccine-associated intussusception cases**								
Rotarix	1558 (1541–1635)	5738 (5570–5906)	530 (515–545)	231 (224–238)	998 (969–1027)	4335 (4210–4460)	5452 (5301–5603)	3893 (3780–4006)
Rotateq	..	..	..	335 (324–346)	1437 (1394–1480)	*..*	*..*	..
Rotavac	1871 (1817–1925)	6903 (6713–7093)	635 (617–653)	278 (270–286)	1200 (1167–1233)	5203 (5060–5346)	6542 (6369–6715)	4690 (4562–4818)
Rotasiil	2345 (2271–2419)	8383 (8125–8641)	758 (735–781)	335 (324–346)	1437 (1394–1480)	6193 (6014–6372)	7841 (7608–8074)	5765 (5583–5947)
Background intussusception	2905	10 197	927	419	1812	7771	9919	7122
Relative incidence of vaccine intussusception*	0·54	0·56	0·57	0·80	0·66	0·56	0·55	0·68
**Vaccine-associated intussusception deaths**								
Rotarix	163 (158–168)	594 (573–615)	11 (11–11)	1 (1–1)	2 (2–2)	90 (87–93)	283 (274–292)	121 (117–125)
Rotateq	..	..	..	1 (1–1)	3 (3–3)	*..*	*..*	..
Rotavac	196 (190–202)	715 (691–739)	13 (13–14)	1 (1–1)	3 (2–3)	108 (105–112)	340 (329–351)	146 (141–151)
Rotasiil	245 (236–254)	864 (834–894)	16 (15–16)	1 (1–2)	3 (3–3)	129 (124–133)	407 (393–421)	179 (173–185)
Cost of managing intussusception associated with the optimal vaccine, US$	$172 656	$498 334	$55 740	$35 975	$122 617	$326 632	$358 415	$396 210
**Total DALYs averted**								
Rotarix	41 213	170 360	1792	193	821	13 241	61 980	35 406
Rotateq	..	..	..	194	810	*..*	*..*	..
Rotavac	39 472	165 114	1638	186	786	12 547	59 540	33 851
Rotasiil	37 044	156 479	1444	175	734	11 638	56 052	31 747
**Average cost-effectiveness ratio (US$ per DALY averted)**								
Rotarix	32·18 (31·84–33·20)	27·53 (26·89–28·10)	445·16 (435·16–450·86)	9292·15 (9133·01–9451·28)	9185·13 (9019·76–9350·50)	306·07 (300·76–311·38)	63·96 (62·85–65·07)	120·12 (118·04–122·19)
Rotateq	..	..	..	18 683·23 (18 363·36–19 003·10)	18 818·21 (18 488·69–19 137·72)	*..*	*..*	..
Rotavac	42·05 (41·30–42·79)	33·03 (32·45–33·61)	477·43 (469·23–485·63)	8598·15 (8448·35–8747·96)	7828·18 (7692·20–7964·16)	364·46 (357·92–371·00)	73·47 (72·16–74·79)	141·30 (138·81–143·80)
Rotasiil	43·94 (43·15–44·72)	36·43 (35·80–37·06)	577·68 (567·30–588·05)	8264·56 (8122·51–8406·60)	7905·07 (7765·92–8044·23)	408·93 (401·73–416·14)	83·03 (81·67–84·49)	160·56 (157·69–163·43)
**Incremental cost-effectiveness ratio to Rotasiil (US$ per DALY averted)**								
Rotarix	Strongly dominates	Strongly dominates	Strongly dominates	19 567 (19 237–19 897)	20 012 (19 625–20 399)	Strongly dominates	Strongly dominates	Strongly dominates
Rotateq	..	..	..	118 524 (116 500–120 548)	124 736 (122 611–126 861)	*..*	*..*	..
Rotavac	Dominates	Dominates	Dominates	13 946 (13 627–14 221)	6740·54 (6650–6832)	Dominates	Dominates	Dominates
Number of fully immunised children with the optimal vaccine	764 567	2 683 476	243 841	110 386	476 785	2 044 984	2 610 286	1 874 277
Total vaccination programme cost with the optimal choice, US$	$1 391 513	$4 900 702	$844 666	$1 526 138	$6 451 612	$4 171 253	$4 187 551	$4 400 808
Total cases of moderate RVGE averted	1 322 569	4 653 291	359 628	156 141	439 069	2 190 424	4 559 525	2 966 937
Total cases of severe RVGE averted	10 119	31 939	2032	899	2158	12 185	22 904	20 488
Averted cases of moderate RVGE seeking health facility care	107 789	379 243	74 623	25 451	71 568	232 185	473 051	307 820
Averted cases of severe RVGE seeking health facility care	3299	10 412	843	293	704	5166	9505	8503
Total RVGE deaths averted	4352	15 644	191	16	73	1355	5872	3174
**Health sector cost of RVGE averted, US$**								
Moderate RVGE	$394 757	$1 388 905	$296 890	$153 477	$431 557	$850 333	$1 732 459	$1 127 332
Severe RVGE	$129 190	$407 736	$35 758	$18 630	$44 762	$202 302	$372 218	$332 979
Total averted health-care cost	$523 947	$1 796 641	$332 648	$172 107	$476 319	$1 052 635	$2 104 677	$1 460 311
**Budget impact, US$**								
Total vaccination cost	$1 391 513	$4 900 702	$844 666	$1 526 138	$6 451 612	$4 171 253	$4 187 551	$4 400 808
Intussusception	$172 656	$498 334	$55 740	$35 975	$122 617	$326 632	$358 415	$396 210
Health-care cost averted	$–523 947	$–1 796 641	$–332 648	$–172 107	$–476 319	$–1 052 635	$–2 104 677	$–1 460 311
Net budget impact	$1 040 222	$3 602 395	$567 758	$1 390 006	$6 097 910	$3 445 250	$2 441 289	$3 336 707
**Total benefits per vaccinated child, US$**								
Rotarix	$22·27	$37·55	$11·96	$27·27	$28·31	$6·97	$8·40	$13·83
Rotateq	..	..	..	$27·38	$27·94	*..*	*..*	..
Rotavac	$21·37	$36·44	$11·06	$26·39	$27·15	$6·64	$8·11	$13·27
Rotasiil	$20·10	$34·57	$9·91	$24·94	$25·43	$6·19	$7·68	$12·49
**Benefit–cost ratio**								
Rotarix	11·36	19·42	3·42	1·66	1·77	3·26	5·07	5·58
Rotateq	..	..	..	0·83	0·87	*..*	*..*	..
Rotavac	8·74	16·15	3·17	1·79	2·06	2·73	4·40	4·73
Rotasiil	8·14	14·42	2·64	1·86	2·03	2·42	3·87	4·12
**Deterministic sensitivity analysis on the optimal vaccine's benefit–cost ratio, lower and upper limits**								
Transition probability of all-cause diarrhoea to death	5·12 and 17·10	10·26 and 29·63	1·26 and 7·22	0·75 and 3·64	0·68 and 4·31	1·35 and 6·08	2·51 and 8·61	3·11 and 9·74
Effectiveness of immunisation	7·15 and 15·41	11·94 and 27·26	2·19 and 5·00	1·20 and 2·62	1·29 and 2·95	2·00 and 4·51	3·27 and 6·98	3·66 and 7·69
Discount rate	8·42 and 13·78	14·42 and 24·78	2·65 and 4·58	1·34 and 2·42	1·47 and 2·69	2·41 and 4·20	3·68 and 6·46	4·12 and 7·06
Cost of vaccine (±25%)	11·90 and 9·99	20·63 and 17·26	3·78 and 3·25	2·22 and 1·55	2·46 and 1·67	3·48 and 2·91	5·46 and 4·53	7·73 and 4·18

Data are n, US$, or (95%CI). DALY=disability-adjusted life-year. RVGE=rotavirus gastroenteritis.

* Relative incidence is the ratio of the incidence within the risk period (week 6 to week 27 in the model) versus the incidence in all other observed period (<52 weeks in the model).

For all the Gavi-eligible countries, the proportion of the probabilistic sensitivity analysis simulations above the BCR threshold ranged from 95·0% (for Comoros) to 100% (for Somalia and South Sudan), which deem the results as robust for all Gavi-eligible countries. For Equatorial Guinea and Gabon, the proportion was 55·8% and 61·3%, respectively. Additional information of the probabilistic sensitivity analysis is available in the appendix (p 2). The deterministic sensitivity analysis showed that the key parameters that can substantially affect the results include the transition probability of all-cause diarrhoea to death, the effectiveness of immunisation, discount rate, and cost of the vaccine. Areas of lower rotavirus mortality rate with higher population will have a higher share of the vaccination impact and vice versa. Further information on the distributional impact is available in the appendix (p 3).

## Discussion

This study provides new evidence to support the decision to introduce the rotavirus vaccine in eight sub-Saharan African countries, using cost–benefit analysis, which has a greater advantage compared with the cost-effectiveness analysis in the allocation of budgets across a range of projects by ranking the projects' BCRs.^34^ The study provides recommendations to support current and future decisions of policy makers in these countries based on the BCR, cost-effectiveness ratio, and budget impact of introducing the rotavirus vaccine. Our findings support the decision to introduce the rotavirus vaccine in the eight sub-Saharan African countries, but with caution in Equatorial Guinea and Gabon. It recommends the use of Rotarix for the six Gavi-eligible countries, but Rotasiil and Rotavac for Equatorial Guinea and Gabon, respectively. The narrow difference in the results of Rotasiil and Rotavac for Equatorial Guinea and Gabon infers that any of the two vaccines can be used in the two countries if conditions do not favour the implementation of the optimal choice for each country.

Compared with previous economic evaluations of the rotavirus vaccine, it is still cost-effective in most Gavi countries and some non-Gavi countries.^35^, ^36^ Although, based on our ICER threshold, the vaccine was not cost-effective in Equatorial Guinea and Gabon, going by the multi-criteria decision analysis framework, the BCR of the vaccine in these countries support consideration for the introduction.^32^ Our results were also similar to findings from a intussusception surveillance study in seven low-income African countries, which showed that the risk of intussusception after vaccination was not higher than the background risk of intussusception.^37^ Due to the low health insurance coverage in sub-Saharan Africa, high cost of managing intussusception, and poverty, a salient question to be addressed by health policy makers in the countries is: who pays for the cost of the vaccine-associated intussusception management, in a case of no child health insurance? This question needs to be addressed especially in low-income countries such as the Central African Republic, Chad, and Somalia.

Our study recommends that introducing the rotavirus vaccine in Equatorial Guinea and Gabon should be initiated with resource-conservative, cautious, and stringent measures. Although the BCR and ICER results in these countries weakly recommend the introduction of rotavirus vaccination, caution must be taken to ensure that the cost is reduced to the barest minimum. Gabon is already planning to introduce the vaccine. An effort needs to be made to ensure that the vaccine is sourced at a very competitive rate and wastage optimally controlled. Equatorial Guinea might need to improve on their poor vaccine coverage rate to maximally benefit from the vaccination programme should they be ready to introduce the rotavirus vaccine.

The introduction of the rotavirus vaccine in non-Gavi countries such as Gabon and Equatorial Guinea might be guaranteed and sustained if rotavirus vaccine manufacturing plants are established in sub-Saharan Africa, which would provide the vaccines at a lower cost for the non-Gavi countries with the aid of the current African continental free trade agreement.^38^ Africa represents about 17% of the world population but has less than 0·1% of the world's vaccine production.^39^ By the year 2050, about 25% of the global population will live in Africa,^39^ which calls for the need to develop more vaccine plants in the continent. With several vaccines development in the pipeline, such as the bovine-human reassortant rotavirus vaccine and human neonatal rotavirus vaccine (RV3-BB) in Indonesia, India, China, and Brazil,^40^ there is hope for a lower price and price sustainability of rotavirus vaccines in the future due to the anticipated market competition.

Our study has some limitations. First, the use of a static Markov model is a limitation when compared with a dynamic model that can capture other diarrhoeal-related outbreaks, such as shigellosis and giardiasis. However, our interest was in rotavirus gastroenteritis, which lessens the effect of this limitation. Second, due to unavailable post-licensure effectiveness data for Rotavac and Rotasiil, we used their efficacy data from phase 3 clinical trials. The results from phase 3 trials could differ from post-licensure effectiveness data, which can affect our study outcomes. There might be a need for further analysis in the future if the post-licensure effectiveness data differ substantially from the efficacy results of the phase 3 trials. Third, the wastage rates used in the assessment were from the WHO vaccine wastage rates calculator. Although we estimated the wastage rates based on the service points and not the WHO target service points computed in the calculator, the wastage rates could vary during implementation due to operational policies. Fourth, we assumed the same waning effect for the four vaccines due to scarce data for the new vaccines, but in practice, the four vaccines waning effect might differ. Fifth, the costs of the vaccines used for the non-Gavi countries were the median prices from UNICEF market survey,^2^ which could vary in these countries based on their negotiation agreement with the manufacturers, should they be ready to introduce the rotavirus vaccine. Sixth, scarce data on the cost of intussusception management in sub-Saharan Africa led to the use of Nigeria intussusception cost data, adjusted for other countries. Using country-specific data would have provided more precise estimates. More importantly, we could not do a quantitative assessment of the distributional effect of the rotavirus vaccines in different regions of each country. A tailored and quantitative distributional impact analysis would have provided the health policy makers in each country with the quantitative effect of the vaccines in their different regions. A quantitative distributional impact assessment would have helped to prioritise resource utilisation in areas with higher BCR results, especially when the country is not ready for a nationwide immunisation programme, but a regional one.

In conclusion, introducing the rotavirus vaccine in the eight countries in sub-Saharan Africa will be a worthwhile decision. Chad, the Central African Republic, Comoros, Guinea, Somalia, and South Sudan should introduce Rotarix, while Equatorial Guinea and Gabon should introduce Rotasiil and Rotavac, respectively. As the BCRs for Equatorial Guinea and Gabon are narrowly above the threshold of 1, and the ICER marginally above the 1 times GDP per capita threshold, stringent measures must be taken to minimise the cost of the introduction and the wastage rate. Although the BCRs of introducing the rotavirus vaccine in these eight countries were above the threshold, the final decision to introduce should be preceded by comparing the BCR of the rotavirus vaccine introduction to the BCRs of other health-care projects.

## Data sharing

The model used in this study was provided to the journal's peer reviewers for their reference when reviewing the manuscript. The data used for the study are provided as an appendix.

## Declaration of interests

CEO reports funding from the Copenhagen Consensus Center, in collaboration with the Bill & Melinda Gates Foundation. OIE declares no competing interests.
