# Supplementary material for: Introducing rotavirus vaccine in eight sub-Saharan African countries: a cost–benefit analysis
Source: Lancet Glob Health. 2021 Jul 21;9(8):e1088–100. doi: 10.1016/S2214-109X(21)00220-5 (PMC8315146; doi:10.1016/S2214-109X(21)00220-5)
Supplement: Supplementary appendix [file mmc1.pdf]

THE LANCET
Global Health

Supplementary appendix

This appendix formed part of the original submission and has been peer reviewed. We post it as supplied by the authors.

Supplement to: Okafor CE, Ekwunife OI. Introducing rotavirus vaccine in eight sub-Saharan African countries: a cost–benefit analysis. *Lancet Glob Health* 2021; **9:** e1088–100.

SUPPLEMENTARY MATERIAL

A. Yearly incidence data from IHME 2019 reports and systematic reviews

Parameters (95% CI)	Countries								
	**Central African Republic**	**Chad**	**Comoros**	**Equatorial Guinea**	**Gabon**	**Guinea**	**Somalia**	**South Sudan**	**Source**
Rate of moderate diarrhoea	2.446 (1.996 – 2.880)	2.858 (2.537 – 3.120)	2.209 (1.740 – 2.670)	1.654 (1.257 – 2.100)	1.794 (1.342 – 2.170)	1.595 (1.267 – 1.980)	2.255 (1.794 – 2.730)	2.455 (1.981 – 2.900)	1
Rate of severe diarrhoea	0.253 (0.184 – 0.339)	0.227 (0.168 – 0.298)	0.185 (0.137 – 0.242)	0.189 (0.075 – 0.375)	0.163 (0.119 – 0.221)	0.186 (0.135 – 0.256)	0.165 (0.123 – 0.219)	0.229 (0.179 – 0.291)	1
Under-1 diarrhoeal death	0.018 (0.009 – 0.027)	0.017 (0.010 – 0.026)	0.003 (0.001 – 0.006)	0.0007 (0.0002 – 0.0015)	0.0013 (0.0005 – 0.0029)	0.0030 (0.0014 – 0.0053)	0.0070 (0.0036 – 0.0121)	0.0059 (0.0028 – 0.0111)	1
1 – 4 years diarrhoeal death	0.003 (0.001 – 0.006)	0.005 (0.002 – 0.008)	0.0005 (0.0002 – 0.0011)	0.00010 (0.00002 – 0.00020)	0.00020 (0.00010 – 0.00050)	0.0009 (0.0004 – 0.0017)	0.0013 (0.0006 – 0.0024)	0.0012 (0.0006 – 0.0023)	1
Recurrent moderate diarrhoea	15.4%								2
Recurrent severe diarrhoea	11.1%								2
Converting rate to probability: $P = 1 - e^{(-rt)}$<br>Converting yearly probability to weekly rate: $Wr = \ln \frac{(1-P)}{52}$									

B. Supplementary data on outcomes of vaccination

Outcomes (95% CI)	Countries							
	**Central African Republic**	**Chad**	**Comoros**	**Equatorial Guinea**	**Gabon**	**Guinea**	**Somalia**	**South Sudan**
Moderate RVGE (MRVGE) without vaccine	3,517,629 (3,498,732 – 3,536,526)	12,503,736 (12,464,762 – 12,541,544)	955,820 (950,130 – 961,508)	419,246 (416,209 – 422,283)	1,180,868 (1,172,833 – 1,188,903)	5,734,852 (5,695,662 – 5,774,042)	12,141,929 (12,067,845 – 12,216,013)	7,920,494 (7,877,141 – 7,963,847)
MRVGE averted with Rotarix	1,322,569 (1,310,307 – 1,334,831)	4,653,291 (4,615,268 – 4,691,314)	359,628 (356,030 – 363,221)	157,141 (153,489 – 159,793)	443,069 (438,337 – 447,801)	2,190,424 (2,167,944 – 2,212,904)	4,559,525 (4,515,490 – 4,603,560)	2,966,937 (2,938,802 – 2,995,072)

MRVGE averted with Rotateq	N/A	N/A	N/A	156,012 (154,357 – 157,667)	446,449 (441,691 – 451,207)	N/A	N/A	N/A
MRVGE averted with Rotavac	1,312,595 (1,300,369 – 1,324,821)	4,629,210 (4,591,388 – 4,667,032)	350,565 (346,983 – 354,147)	156,110 (154,465 – 157,755)	439,069 (434,374 – 443,764)	2,141,314 (2,119,028 – 2,163,600)	4,524,452 (4,480,936 – 4,567,968)	2,944,759 (2,916,947 – 2,972,571)
MRVGE averted with Rotasiil	1,333,177 (1,320,077 – 1.346,277)	4,575,906 (4,538,229 – 4,613,583)	346,437 (342,437 – 349,619)	156,141 (154,489 – 157,793)	436,002 (431,304 – 440,700)	2,148,059 (2,125,579 – 2,170,539)	4,469,765 (4,426,264 – 4,513,266)	2,921,290 (2,893,619 – 2,948,961)
Severe RVGE (SRVGE) without vaccine	17,167 (16,976 – 17,358)	53,680 (53,166 – 54,194)	3,390 (3,354 – 3,426)	1,497 (1,457 – 1,537)	3,716 (3,670 – 3,762)	20,485 (20,235 – 20,735)	38,410 (37,979 – 38,841)	34,766 (34,440 – 35,092)
SRVGE averted with Rotarix	10,119 (9,984 – 10,254)	31,939 (31,562 – 32,316)	2,032 (2,006 – 2,058)	908 (883 – 933)	2,189 (2,157 – 2,221)	12,185 (12,013 – 12,357)	22,904 (22,601 – 23,207)	20,488 (20,248 – 20,728)
SRVGE averted with Rotateq	N/A	N/A	N/A	890 (865 – 915)	2,209 (2,177 – 2,241)	N/A	N/A	N/A
SRVGE averted with Rotavac	10,064 (9,847 – 10,281)	31,445 (31,070 – 31,820)	2,017 (1,991 – 2,043)	898 (873 – 923)	2,158 (2,126 – 2,190)	12,070 (11,899 – 12,241)	22,562 (22,261 – 22,863)	20,398 (20,160 – 20,636)
SRVGE averted with Rotasiil	10,001 (9,787 – 10,215)	31,166 (30,794 – 31,538)	1,962 (1,936 – 1,988)	899 (874 – 924)	2,165 (2,133 – 2,197)	12,127 (11,955 – 12,299)	22,349 (22,050 – 22,648)	20,231 (19,995 – 20,467)
RVGE deaths without vaccine	10,705 (10,559 – 10,851)	39,163 (38,709 – 39,617)	475 (465 – 485)	42 (41 – 43)	183 (179 – 187)	3,302 (3,244 – 3,360)	14,611 (14,359 – 14,863)	7,862 (7,718 – 8,006)
RVGE deaths averted with Rotarix	4,352 (4,290 – 4,414)	15,644 (15,454 – 15,834)	191 (187 – 195)	16 (16 – 17)	74 (72 – 76)	1,355 (1,331 – 1,379)	5,872 (5,766 – 5,978)	3,174 (3,114 – 3,234)
RVGE deaths averted with Rotateq	N/A	N/A	N/A	18 (17 – 18)	77 (75 – 79)	N/A	N/A	N/A
RVGE deaths averted with Rotavac	4,238 (4,178 – 4,298)	15,325 (15,140 – 15,510)	187 (183 – 191)	17 (17 – 18)	73 (71 – 75)	1,314 (1,291 – 1,337)	5,721 (5,620 – 5,822)	3,122 (3,064 – 3,180)
RVGE deaths averted with Rotasiil	3,993 (3,943 – 4,043)	14,795 (14,615 – 14,975	181 (177 – 185)	16 (16 – 16)	70 (68 – 72)	1,345 (1,321 – 1,369)	5,510 (5,414 – 5,606)	3,004 (2,948 – 3,060)
Children immunised with Rotarix	764,567±20	2,683,476±50	243,841±12	110,411±10	476,892±15	2,044,982±43	2,610,286±38	1,874,277±32
Children immunised with Rotateq	N/A	N/A	N/A	110,386±9	476,785±14	N/A	N/A	N/A
Children immunised with Rotavac	764,067±20	2,681,989±48	243,749±10	110,386±9	476,785±15	2,044,144±43	2,608,715±37	1,873,485±31
Children immunised with Rotasiil	764,063±20	2,681,972±48	243,748±11	110,386±9	476,785±14	2,044,143±43	2,608,709±37	1,873,481±31
YLD without vaccine	347 (341 – 353)	1,676 (1,656 – 1,696)	117 (115 – 119)	51 (50 – 52)	137 (135 – 139)	728 (719 – 737)	1,671 (1,648 – 1,694)	1,043 (1.030 – 1,056)
YLL without vaccine	81,499 (79,926 – 83,072)	441,707 (451,192 – 462,222)	5,660 (5,545 – 5,775)	464 (452 – 476)	2,055 (2,006 – 2,104)	36,600 (35,960 – 37,240)	169,085 (166,167 – 172,003	95,142 (93,392 – 96,892)
PSA below ICER threshold	99.7%	100%	95%	44%	40%	99%	100%	100%

C. Additional description of some terms, analysis, and outcomes

1. Gavi-eligible countries refer to countries whose three-year average gross national income (GNI) per capita are below or equal to the eligibility threshold (US$1,630).[3] They include low-income countries (LIC), also referred to as countries in the initial self-financing phase, and countries in the preparatory phase 1.[3] Gavi financial support for preparatory phase 1 countries decreases annually as per Gavi policy for phase 1 countries.[3] Phase 2 countries GNI per capita are above the eligibility threshold and the financial support they receive from Gavi decreases annually (until they fully self-finance the vaccines) as per Gavi policy for phase 2 countries.[3] Phase 3 countries are fully self-financing countries but commit with vaccine manufacturers to procure vaccines at the price Gavi pays under specific conditions and period.[3] Non-Gavi countries have crossed phase 3, receive no support from Gavi, and have no commitment to procure vaccines at the Gavi price from vaccine manufacturers.

2. The wastage cost is the cost associated with vaccine damages or losses during transportation, storage, or administration, which has an impact on the total vaccine cost and total immunisation delivery cost. The vaccine wastage cost is the cost of the vaccine multiplied by the wastage rate, whilst the cold-chain volume wastage cost is the cost of the cold chain volume multiplied by the wastage rate.

3. The cost estimates we used from the ICAN review included personnel costs and logistics costs related to immunisation delivery. To estimate the immunisation delivery cost for LIC in this study, we used the cost estimate for Rwanda from the ICAN review, estimated for Rotateq. For Comoros, we used Rotarix estimates for Zambia and Ghana (lower-middle-income countries), whilst for Equatorial Guinea and Gabon (upper-middle-income countries), we used Rotarix estimate for Colombia. We adjusted the costs data obtained from the ICAN review to the cost for each country in this study using their relative price-level ratios.[4] From the ICAN review, a relative vaccination coverage ratio of ‘2’ between countries of the same income classification and population size was associated with a 9% to 15% increase in immunisation delivery cost.[5] This vaccine coverage factor was applied to adjust the cost data of the eight countries being evaluated. We then adjusted the costs based on the number of doses for each vaccine and their waste-adjusted cold-chain volume. The cold-chain volume contributed to about 7%, 10%, 12%, and 16% of the total immunisation delivery cost for Rotavac, Rotarix, Rotasiil, and Rotateq respectively, from our adjustment.[5] The costs were adjusted to 2019 USD as per guidelines of the CCEGM and the EPPI-Centre.[6]

4. Distribution of impact:

   Most remote areas in each country in SSA have a higher RVGE burden compared to the urban areas.[1] These areas are also associated with a higher cost of healthcare due to shortage of health professionals, poor healthcare-seeking and access to care.[7] Thus, it is highly probable that these areas will have a lower share of the RV vaccination impact at the patient level. However, at the population level, if the remote areas have a relatively higher population than the urban areas, they will benefit more from the vaccination program compared to urban areas.

References

1 Institute for Health Metrics and Evaluation. Global Burden of Disease Study 2019 (GBD 2019) Results. Seattle, United States: 2019. http://ghdx.healthdata.org/gbd-results-tool (accessed July 11, 2020).

2 Lamberti LM, Fischer Walker CL, Black RE. Systematic review of diarrhea duration and severity in children and adults in low- and middle-income countries. *BMC Public Health* 2012; **12**: 276.

3 Gavi. Eligibility and transition policy. 2020. https://www.gavi.org/types-support/sustainability/eligibility (accessed Oct 10, 2020).

4 WorldBank. Indicators: Economy & Growth. 2019. https://data.worldbank.org/topic/economy-and-growth?view=chart (accessed Sept 10, 2020).

5 Immunization Costing Action Network (ICAN). Immunization Delivery Cost Catalogue. 2019. http://immunizationeconomics.org/ican-home (accessed June 5, 2020).

6 CCEMG and EPPI-Centre. CCEMG - EPPI-Centre Cost Converter v.1.6. 2019. https://eppi.ioe.ac.uk/costconversion/default.aspx (accessed March 15, 2020).

7 WHO. Global Health Workforce statistics. 2019. https://www.who.int/data/gho/data/themes/topics/health-workforce (accessed Oct 9, 2020).
